# Monitoring population and environmental parameters of invasive mosquito species in Europe

**DOI:** 10.1186/1756-3305-7-187

**Published:** 2014-04-16

**Authors:** Dušan Petrić, Romeo Bellini, Ernst-Jan Scholte, Laurence Marrama Rakotoarivony, Francis Schaffner

**Affiliations:** 1 University of Novi Sad, Faculty of Agriculture, Laboratory for Medical Entomology, Trg D. Obradovića 8, 21000 Novi Sad, Serbia; 2 Centro Agricoltura Ambiente "G. Nicoli", Via Argini Nord 3351, 40014 Crevalcore, Italy; 3 National Centre for Monitoring of Vectors, Dutch Food and Consumer Product Safety Authority (NVWA), Geertjesweg 15, P.O. Box 9102, 6700, HC, Wageningen, The Netherlands; 4 ECDC, European Centre for Disease Prevention and Control, Tomtebodavägen 11A, 17183 Stockholm, Sweden; 5 Avia-GIS, Risschotlei 33, B-2980 Zoersel, Belgium

**Keywords:** Invasive mosquito, Population size, Blood feeding behaviour, Dispersal, Environmental change, Land use, Vector, Europe, Risk assessment, Surveillance

## Abstract

To enable a better understanding of the overwhelming alterations in the invasive mosquito species (IMS), methodical insight into the population and environmental factors that govern the IMS and pathogen adaptations are essential. There are numerous ways of estimating mosquito populations, and usually these describe developmental and life-history parameters. The key population parameters that should be considered during the surveillance of invasive mosquito species are: (1) population size and dynamics during the season, (2) longevity, (3) biting behaviour, and (4) dispersal capacity. Knowledge of these parameters coupled with vector competence may help to determine the vectorial capacity of IMS and basic disease reproduction number (R_0_) to support mosquito borne disease (MBD) risk assessment. Similarly, environmental factors include availability and type of larval breeding containers, climate change, environmental change, human population density, increased human travel and goods transport, changes in living, agricultural and farming habits (e.g. land use), and reduction of resources in the life cycle of mosquitoes by interventions (e.g. source reduction of aquatic habitats). Human population distributions, urbanisation, and human population movement are the key behavioural factors in most IMS-transmitted diseases. Anthropogenic issues are related to the global spread of MBD such as the introduction, reintroduction, circulation of IMS and increased exposure to humans from infected mosquito bites. This review addresses the population and environmental factors underlying the growing changes in IMS populations in Europe and confers the parameters selected by criteria of their applicability. In addition, overview of the commonly used and newly developed tools for their monitoring is provided.

## Introduction

Invasive mosquito species (IMS) are defined by their ability to colonize new territories. Human activity, especially the global movement of trade goods, has led to the passive dispersion of species previously confined to specific regions. A considerable increase in the spread of IMS has been observed within Europe since the late 1990s, with the Asian tiger mosquito *Aedes albopictus* [*Stegomyia albopicta*] having continuously expanded its distribution and several other container-breeding *Aedes* species being reported from new countries almost every year [1]. It is estimated that presently 45% of the total human population of Europe is exposed to the risk of IMS and pathogens they could transmit [2].

Mosquitoes may be of public health relevance either when they occur in high densities and cause a nuisance or when they transmit disease agents. Over recent decades, human contact with mosquitoes has become more frequent as peri-urban suburbs expanded into previously undisturbed natural areas, thus providing a greater number and variety of mosquito breeding places than inner-city areas. In addition, urbanised areas are facing an invasion of container-breeding mosquitoes such as *Ae. albopictus* which is an aggressive nuisance biter during the day when females are seeking blood meals from humans. Invasive mosquitoes are often also putative or efficient vectors of pathogens as demonstrated by the recent outbreaks of chikungunya and dengue fevers in the Mediterranean, caused by *Ae. albopictus* (which in addition is competent to transmit at least 22 arboviruses) [3-9].

Once established, the success of IMS in reproducing and spreading will be governed by a complex range of intrinsic population factors (e.g. longevity, host searching behaviour) and extrinsic environmental and anthropogenic parameters (e.g. climate, human population movements, travel and trade). These factors affect the interactions between pathogens, vectors and hosts, including humans, making investigations on mosquito borne diseases (MBD) a composite task.

The present article aims to investigate the key population and environmental parameters, and to discuss their importance and currently available procedures of data collection, in the frame of surveillance of IMS in Europe, as described and promoted in The European Centre for Disease Prevention and Control (ECDC) guidelines [10].

## Review

### Mosquito population parameters

Mosquito population parameters are all mosquito-related, i.e. parameter values are primarily dependent on the IMS, which adapts to its new environment, whereas environmental parameters are determined by the environmental and climatic conditions, which have an impact on the mosquito population. If possible, it is always beneficial to assess them initially and immediately, when IMS populations become established and before local MBD transmission has commenced.

Most difficulties and complications in controlling IMS and MBD arise from a lack of information from the beginning, in particular as answers to the following questions: What are the pathways for disease/vector introduction from abroad? Is there a mosquito population that can transmit a certain pathogen present in the country? How abundant is the putative vector mosquito? Do these mosquitoes prefer to blood-feed on humans or animal host? Do they imbibe on different/multiple hosts before being completely fed? When do they search for a blood meal? How efficiently can they transmit disease agents within human populations?

Population estimates of IMS usually describe a wide range of species-specific developmental life-history parameters that are affected by the characteristics of their new environment. They include: abundance; longevity; the intrinsic rate of increase; the net reproductive (replacement) rate; birth rate; death rate; generation time; the number of gonotrophic cycles in a mosquito’s lifetime and their length; fecundity; fertility; host preference; capacity for dispersal, and size of population (Table 1). Indeed, the life cycle of female mosquitoes requires that physiological needs like sugar meal, mates, blood meal, resting places, and oviposition sites are met and satisfied. Thus, the comparison of life-history strategies of invasive container-breeding mosquitoes may yield insights into the factors that permit certain species to expand their geographical range.

**Table 1:** **Population parameters of IMS**^**1**^**, a dictionary**

**Parameter**	**Meaning**
Abundance	Strictly applicable to quantity only; number of specimens of a certain species (absolute, relative, or index)
Basic reproduction number R_0_	The average number of secondary cases of disease arising from each primary infection in a certain population of susceptible humans/hosts. The disease can invade/maintains if R_0_ > 1, whereas it cannot/decreases if R_0_ < 1.
Birth rate	The ratio of the number of live births in a period of time in a given area/larval habitat in relation to a given portion of the population in that area/larval habitat.
Biting behaviour	Usually related to host finding (foraging) behaviour of the species. Most often depicts the part of the day (diurnal, crepuscular, nocturnal biting behaviour) when most of the specimens of a particular mosquito species forage for the blood meal.
Blood feeding behaviour	Haematophagy (sometimes spelled hematophagy) is the practice of mosquitoes of feeding on blood. In relation to host preference, mosquitoes could be opportune (specialized) or catholic (unspecialized) feeders. If IMS tends to feed repeatedly (from same or different host) to complete one blood meal it is called multiple feeding.
Death rate	The frequency of death; the proportion of deaths in a specified number of the population (mortality rate).
Density	The number of individuals of the same species that live in a given unit area.
Dispersal	The outward extension of the range of the species, usually resulting from the chance event; ability of an IMS to spread around/from the breeding site.
Fecundity	The innate capacity of an organism to form reproductive elements such as ova or sperm; the potential capacity for reproduction.
Fertility	The natural capability to produce offspring; as a measure, "fertility rate" is the number of offspring born per mating pair, individual or population.
Generation time	The doubling time of a species under the influence of certain ecological conditions, or the time elapsed from one egg laying to the next.
Gonotrophic cycle	The duration of time between two ovipositions, i.e. the time females spend for host-seeking, blood feeding, resting (digestion and egg maturation time), and oviposition (seeking the site and laying the eggs) in nature, or from blood meal to egg laying in the laboratory.
Intrinsic rate of increase	A population’s growth rate, derived by subtracting the instantaneous death rate from the instantaneous birth rate (innate rate of increase).
Longevity	The duration of life of an individual (lifespan).
Net reproductive (replacement) rate	The total amount of offspring that a newly born female can expect to bear during a lifetime.
Survival rate	The rate of specimens remaining alive in a given period of time (e.g. daily), especially under adverse conditions.
Vectorial capacity	A mathematical expression of the probability of disease transmission by a specific vector species. The average number of inoculations from a single case of disease in a day, from vector population to man, assuming that all mosquitoes that bite an infected person get infected. This is mosquito component of the basic reproduction number R_0_.
Vector competence	Ability of a mosquito species to transmit a specific disease expressed in relative number of females infective (usually head or salivary glands are checked) with the pathogen.

^1^Estimates of these parameters are available in the literature for most of the vector species. However, it is recommended to also estimate them for the local mosquito population as these parameter estimates might vary according to the population and are influenced by environmental factors.

With regard to IMS, population parameters help to detect IMS early and before they can spread from the site of introduction [11]. The life history of the mosquito species is also of crucial importance for estimating its vectorial capacity, interpreting trap data, assessing the risk of MBD transmission and modelling potential outbreaks and disease spread. Moreover, understanding of population parameters supports the development of effective control programmes and the evaluation of their impact [12]. It may also help to establish efficient mass rearing facilities for the sterile insect technique (SIT) to be used as a tool within integrated control programmes and to evaluate the impact of sterile insect release [11,13].

In addition, evidence is accumulating to suggest that changes may be occurring in fluctuating local mosquito populations and the population parameters of different cohorts of a species may be quite different [13-18]. In some cases, fluctuation of the vector population parameters might be linked to the acclimation of a population to abiotic factors. These changes need to be determined to better understand the dynamics of the vectorial capacity, especially in countries with a wide range of temperatures (exhibiting different climates and covering a broad altitudinal range). Therefore, parameters such as fertility, longevity and vectorial capacity should be estimated in the laboratory (e.g. simulating the influence of different temperatures) and continuously monitored in the field.

The key population parameters to be considered for the surveillance of IMS are: (1) population size and dynamics during the season, (2) longevity, (3) biting behaviour, and (4) dispersal capacity (Table 2). Indeed these parameters combined with vector competence may help to determine the vectorial capacity and to provide a basis for MBD risk assessment.

**Table 2:** Main characteristics of key population parameters of IMS

**Parameters**	**Information provided**	**Strengths**	**Weaknesses**	**Data collection methods and equipment**
IMS abundance	Quantitative estimation of IMS adult population; seasonal dynamic; comparative analysis throughout the years; nuisance and MBD risk assessment	Supports the evaluation of nuisance threshold definition, specific risk assessment and control efforts	Good organization and quality control required	Breeding site inspections; ovitrap, pupal, or adult surveys
				Adequate field material
Female longevity, gonotrophic cycle and dispersal	Life traits key parameters required to evaluate MBD risk	Valuable data to feed epidemiological equation	High-tech laboratory required; large spatio-temporal variability; needs replications; expensive	Field mark-release-recapture; laboratory experiments
				Rearing facilities and special equipment
Female biting behaviour	Life traits key parameter required to evaluate MBD risk; nuisance protection; nuisance threshold	Valuable data to feed epidemiological equation; inform citizen	Needs high tech laboratory; extensive field work; high cost	Field and laboratory experiments
				Traps and laboratory equipment
Population vector competence	Life traits key parameters required to evaluate MBD risk for main pathogens	Essential data to feed epidemiological equation	Needs BL3 laboratory; expensive	Laboratory infections
				BL3 rearing facilities and equipment

### Population size

Population size estimates can be expressed as absolute or relative, and in the form of population indices. For most animals, numbers of absolute estimates are expressed as a density per unit area or volume (absolute population) or density per unit of the habitat, e.g. per water volume or per host (population intensity) [19]. In relative estimates, the numbers sampled cannot be expressed as density or intensity per area or habitat unit, and can only be used to compare data in space or time. Relative estimates are especially useful in assessing the species’ relative density, dispersal, distribution, and host preference. If mosquitoes are not counted, but number of their occurrence in breeding sites is recorded, the resulting estimate is a population index.

There is no clear-cut division between relative and absolute methods of sampling. Absolute methods are rarely 100% efficient. Both relative estimates and population indices can sometimes be related to absolute population when sufficient data are obtained and measured at the same time.

Relative methods are important in applied areas, such as IMS and MBD surveillance programmes, where most of the information available may be derived from eggs, larvae and pupae sampling and adult trapping. Even relative abundance is often difficult to calculate requiring statistically based sampling design, adequate sampling equipment and stable financial support [20].

Hence, it is a sound practice to estimate the population and environmental parameters by more than one method. In the long run, more knowledge of the ecology of the insect may be gained by studying new areas, using other techniques, or taking further samples instead of struggling for a very high level of accuracy in each operation [19].

The indices traditionally used to evaluate *Stegomyia* population (e.g. *Ae. aegypti* [*St. aegypti*], *Ae. albopictus*) densities and the efficacy of control campaigns, such as the house index (HI: percentage of houses with at least one active breeding site), the container index (CI: percentage of containers with larvae), the Breteau index (BI: number of active breeding sites per 100 premises), and the ovitrap index (OI: the average proportion of ovitraps with mosquitoes) are widely used as standard empirical parameters in developing countries [21-24]. The same indices could also be potentially applied to other IMS with similar oviposition habits, such as *Ae. atropalpus* [*Georgecraigius atropalpus*], *Ae. japonicus* [*Hulecoeteomyia japonica*], *Ae. koreicus* [*Hl. koreica*], and *Ae. triseriatus* [*Ochlerotatus triseriatus*].

However, the traditional indices used to evaluate *Stegomyia* populations (CI, HI, BI, OI) have some disadvantages when implemented in epidemiological studies [25]. The CI only considers the percentage of positive containers and not their absolute number (either per unit area, per premise, or per person). The HI is more accurate than the CI because it refers to the number of houses, but it is again limited because it does not account for the number of positive containers. The BI is the only index that combines data on positive containers with the density per premise [21]. The main limitation of the three indices is the lack of information referring to the real productivity (amount of individuals over a given period of time expressed as a unit/time rate) of the containers, the way these indices describe the relation to the adult population size, and their applicability to the larger European cities [26].

Indeed, results obtained using these indices are of limited value in European countries because of the differences in socio-economic and structural conditions that characterise human dwellings and the differences in the availability of breeding sites in public areas. Other indices that are more appropriate for European urban areas, devised from pupal demographic surveys (PDS) are the PPI (number of pupae/premise) and PHI (number of pupae/hectare), which define the mosquito density per unit area, applicable to both public and private domains. The PDS exploits the strong correlation between the number of pupae and the number of adults in a defined area, based on the low natural mortality usually affecting the pupal stage [26].

Studies on the correlation between traditional indices and adult population densities show contradictory results: while some evidenced a good correlation between BI and both the larval and the adult densities [27], others found no correlation between traditional indices and the PHI or pupae per person (PPP) [28]. In a recent study conducted in Italy, a statistically significant correlation between PHI and the mean number of eggs/ovitrap was found [29]. Similarly, authors correlated the number of females/hectare, estimated on the basis of the number of sampled pupae, with the number of eggs. Finally, they suggested that the number of eggs estimated by means of ovitrap monitoring can be used to determine the mean number of biting females per unit area. Trap positivity index (TP: the proportion of positive traps) and an egg density index (MED: the ratio between the total number of eggs collected and the total number of traps) were used to compare differences between seasons per neighbourhood and to produce infestation maps [30]. It should be mentioned that sample size is of crucial importance for obtaining reliable data [29]. A particular method was developed to be used in surveys of mosquito pupae, for identifying the key container types producing the majority of adult dengue vectors. A step-wise rule, based on the entropy of the cumulative data, was devised for determining the number of houses positive for pupae, at which a pupal survey might reasonably be stopped [31].

The transmission thresholds for dengue based on the standing yield of *Ae. aegypti* PPP were developed for use in the assessment of risk of transmission and to provide targets for the actual degree of suppression by type of breeding container required to prevent or eliminate transmission in source-reduction programmes [32]. When coupled with field observations from PDS, it was possible for the first time to know the relative importance of the various types of containers in contributing to the vector population [25,26,32].

Under conditions prevailing in the 2007 chikungunya outbreak area in Italy, positive correlation was found between the female density estimated by means of PDS, human landing collection (HLC), number of bites per citizen (NBC) and the mean number of eggs in the ovitraps [33]. Reproduction number (R_0_) calculated out from the number of biting females estimated from the egg density was comparable to the basic disease R_0_ calculated based on the progression of the human cases [33]. The identification of an epidemic threshold based on the mean egg density mentioned might be useful in defining risk areas, risk seasonal periods and better planning control programmes.

### Longevity

In order to estimate the longevity of a mosquito population, one needs to collect absolute data or convert relative values. Consequently, this type of research can be done in the laboratory, by rearing the target species [14,17,34-38], or in the field [39], typically deriving the data from mark-release-recapture (MRR) trials [13,15,18,40-46]. Some authors combine the advantages of controlled and natural environments and set up their experiment in semi natural/uncontrolled conditions [11,16]. Within a given population of mosquitoes, for example, both vectorial capacity and the extent to which the potential fecundity is realised is influenced by the longevity of the females. The influence of temperature and other environmental factors that are studied in the laboratory can then be applied in the field but only to the same populations from which they were derived [47].

The most frequently used factors when estimating the longevity of adult mosquitoes in the laboratory are water, blood meal, sugar solution availability [38,42,48] and temperature [37]. Even though, larval density affects size and longevity directly [19], the impact of forecasted rising temperatures on larval development and longevity is less obvious. Rising temperatures can speed up larval development but also lead to a reduction in the body size of juveniles and hence reduce adult longevity. Differences in body size of adult mosquitoes can influence the vectorial capacity. Large *Ae. albopictus* females have higher human host attack rates and obtain multiple blood meals (from multiple hosts) more frequently than small females [38], thus potentially spreading the disease more efficiently. However, the number of blood meals and the frequency of host-seeking behavior were negatively correlated with body size in the *Ae. albopictus* Nagasaki strain [49]. Body size may also affect mosquito survival and longevity under natural conditions which is of paramount ecological importance because longevity affects net reproduction rates and dispersal distance [50]. Still, the impact of temperature, nutrition level and genetics may affect general growth rule “hotter is smaller”. Contrary to this rule, higher temperatures can result in producing mosquitoes with shorter wings and greater body mass, and effects of temperature could be dependent upon available food and mosquito strain [49,51].

The MRR method is frequently used to estimate longevity, population size and dispersal. As for the population size, if a sample from a population (captured specimens) is marked, returned to the original population, and then, after complete mixing, re-sampled, the number of marked individuals in the recapture sample will have the same ratio to the total number in the second sample as the total of marked individuals originally released have to the total population. Time or spatial scales are applied to estimate longevity or dispersal. A basic prerequisite for the use of this method is a technique for marking the animals so that they can be released unharmed and unaffected into the wild and recognised again on recapture [19]. Fluorescent dyes are the most widely used marking technique in mosquito MRR experiments [13,18,41,46] but recently rubidium (Rb)-marked blood [15] or a mosquito strain whose natural infection of *Wolbachia* had been removed [13] or induced [52] have been employed as well.

### Blood feeding behaviour

Investigations of mosquito blood feeding and resting behaviour are of crucial importance for areas where epidemics occur and usually comprise the investigation of host-seeking and feeding behaviours on several vertebrate species, the measuring of endophagous/exophagous biting behaviour, endophilic/exophilic resting behaviour, and the mosquito’s daily biting activity (recorded over 24 hours). *Aedes albopictus* prefer to feed (89% exophagic) and rest (87% exophilic) outdoors [53] in contrast to *Ae. aegypti*, which is well-adapted to the highly urban environments of tropical cities and frequently bites and rests indoors [54].

Blood-feeding behaviour can influence vector potential, depending on the vertebrate host groups which the mosquito makes contact with. If reservoir and amplifier hosts (in which pathogen multiplies) are the primary focus of vector blood-feeding, the likelihood of pathogen acquisition by the vector increases [55]. Also, transmission probability would be much higher if seasonal and circadian biting activity of vector overlaps the behaviour of its host [56]. Mosquitoes can be opportunist, feeding on a wide range of cold- and warm-blooded hosts. Such mosquito species could be a potentially bridge vector of zoonotic pathogens to humans (e.g. West Nile virus), but in contrast is likely to be less efficient as an epidemic vector of pathogens restricted to humans (e.g. dengue, chikungunya viruses) [54]. Conversely, anthropophily (preference for humans) combined with multiple blood feeds during completion of one meal, increases the risks of spreading an arbovirus within the human population. This scenario might be further complicated in the future by the introduction of new IMS competent to transmit the same pathogen but showing a complementary dial biting activity in respect to the indigenous vector (e.g. introduction of *Ae. albopictus* in an area where *Cx. p. pipiens* already transmits *Dirofilaria immitis* and *D. repens*) [55]. Prevalence of microfilaremic dogs and presence and abundance of competent vectors also affect the rate of infestation within a given mosquito population, which, in turn, is directly related to the risk for a native dog to be infested [57]. Therefore, knowledge of the biological parameters that lead to host choice can be highly relevant for the planning of mosquito and MBD control [57,58].

Host preference and blood feeding behaviour can be assayed outdoors or in the laboratory [59] using olfactometer or cages of various construction and various hosts [60]. Using humans as a host is very important in the study of mosquito attractants, repellents, and host preference. However, mosquito bites cause potential medical problems because of hypersensitivity and perhaps secondary bacterial infection, even when using laboratory mosquitoes. Moreover, once a female mosquito has fed on human blood, it cannot be used in subsequent probing tests. Solution to these problems is offered by introducing a proboscis (mosquito mouth part) amputation technique [60].

Host-preference experiments conducted outdoors are based on host-baited traps of various design [61-63], odour-baited traps [64,65], or on blood meal analysis [66-68]. Hosts of blood-fed mosquitoes can be identified with an indirect enzyme-linked immunosorbent assay by using antisera made in rabbits for sera of animals that would commonly occur in certain habitats. Blood meals taken from birds can e.g. be identified to species by a PCR-HDA [69]; blood meals from humans (including multiple blood meals taken from more than one human) can be identified by STR/PCR-DNA profiling technique, which involves amplification of three short tandem repeat loci [68,70,71]. A universal DNA barcoding and high-throughput diagnostic tool for identification of vertebrate host from arthropod blood meals was recently provided [72]. Appropriate methods for data processing, host feeding patterns, and host feeding indices calculation should also be considered [68].

Assessing the nuisance thresholds for dominant mosquito species is of a great value for the evaluation of conventional control measures [73,74] but estimation of a disease transmission threshold needs intensive sampling and expert data processing of, for example, the PPP. As for disease, it usually involves deciding which seasonal estimates to use, what temperature to use, and what value for overall seroprevalence of virus antibody to use [32].

Gonotrophic cycle (the time females spend from finding a host to laying the eggs in nature, or from blood meal to egg laying in the laboratory) is another population parameter connected both with host-finding and blood-feeding but also with resting, digestion of blood, oocyte maturation and oviposition. Its duration determines how many hosts a female will be feeding during its lifetime, which greatly influences the chances of finding an infectious host and transmitting a pathogen. The length of the gonotrophic cycle under natural conditions could be divided into three parts: (a) the time spent for host-seeking, i.e. starting with a blood meal in laboratory [75]; (b) resting, i.e. digestion and egg maturation time, and (c) oviposition time for seeking the site [76]. The frequency of mosquitoes biting humans is estimated as the ratio of the human blood index (HBI) to the length of the gonotrophic cycle [76].

### Dispersal

Flight ability, flight ranges and dispersal capacity are the parameters indicating the distance that mosquitoes are able to travel (actively, by itself; or passively, by human transportation) from their breeding places to search for sugar meal, mates, blood meal, resting places and oviposition sites. Dispersal to seek a host is epidemiologically important as it influences the capacity of female mosquitoes to acquire and disseminate pathogens. Dispersal for oviposition is also relevant to disease transmission as it increases dispersal of potentially infected progeny [14]. Better methods of IMS sampling to evaluate the movements of adult mosquito vectors in endemic or epidemic areas in Europe are needed to estimate disease transmission dynamics and to define the areas where to implement vector control measures [65,77].

The dispersal flight of mosquitoes is influenced by factors such as blood sources density and distribution, availability of oviposition sites, weather (e.g. wind, RH, temperature, rainfall), terrain features, vegetation, housing characteristics in urban environments [15,18,78,79] and species-specific traits.

During the early period of dispersal of *Ae. albopictus* in the USA, its presence appeared to be related to the proximity to interstate highways [80]. The postulated relationship between dispersal and major transportation routes would be expected for all IMS transported largely by human activities, such as the commercial movement of used tyres for retreading (recapping) or recycling [81], ornamental plant trade, and individual, public and commercial transportation from infested areas. Once an IMS becomes established, local transport and active dispersal may make possible the rapid colonization throughout the surrounding area and sometimes even to remote regions [80,82,83].

Estimations of active mosquito dispersal are most frequently carried out by means of MRR studies, the effectiveness of which is strongly affected by the quantity of marked specimens released and the ability to carry out recapture over a large enough study area [50]. The availability of an effective recapture method may represent a serious limitation in MRR studies.

In addition, results obtained from MRR experiments cannot be generalised because they greatly depend on the ecological characteristics of the study sites. And, the ecological factors affecting dispersal may vary depending on the objectives of the mosquito dispersion (i.e. host seeking, resting or oviposition site seeking), which in turn, implies different recapture approaches. Inconsistent results obtained in Australia, Brazil and Italy [15,18,44,84,85] emphasise the importance of evaluating the dispersal capacity at local levels. Preferably, surveys should be conducted for all host seeking, resting and ovipositing females, and also for males if SIT is going to be implemented.

Mosquito behaviour can strongly influence trapping results, e.g. some species of mosquitoes may fly close to the ground while searching for a blood meal, whilst others do not (Petrić *et al*., unpublished observations). In the case of endophilic species, marked mosquitoes can be efficiently recaptured by active aspiration in houses during their indoor resting phase [86], but this approach is much less efficient for collecting exophilic mosquitoes resting outdoors [87]. Mouse-baited traps were used to assess the longevity and dispersal of male and female *Ae. albopictus* by MRR [45]. Females could be fed with rubidium-marked blood and afterwards detected Rb in ovitrap-collected eggs by atomic emission spectrophotometry [15]. For investigating the dispersal of *Ae. albopictus* males in urban localities by MRR techniques, recapturing of the *Wolbachia* free males on human hosts and while swarming has been employed in northern Italy [13]. The mean distance travelled for *Wolbachia*-free males was significantly higher than for males marked with fluorescent powder. In the same paper authors characterised the dispersal pattern by mean distance travelled (MDT), maximum distance travelled (MAX), and flight range (FR), and presented useful procedures for data processing. High recapture rate of 4.3% was also obtained by using sticky traps in MRR experiments to study the dispersal of *Ae. albopictus* females in Rome, Italy [18]. BG Sentinel traps (Biogents, Regensburg, Germany) were used to estimate the size of adult *Ae. aegypti* populations by release of adults infected with *Wolbachia* into uninfected *Ae. aegypti* populations around Cairns in far north Queensland, Australia [52]. Traditionally, CDC backpack aspirators are used for recapturing resting females [84,88]. Other adult traps, like Gravid *Aedes* Trap (prototype) and MosquiTRAP (Ecovec Ltd., Belo Horizonte, Brazil), and sticky traps are capturing oviposition-seeking females [18,44,84,88-90], while the BG-Sentinel trap mainly samples host-seeking females [84,91].

### Environmental parameters

In addition to species specific population factors of particular mosquito species, environmental factors play an important role in determining the IMS’ colonisation process, its population size, its vectorial capacity, and consequently the MBD transmission risk. Such factors include (1) availability and type of larval breeding containers, (2) climate change, (3) environmental change, (4) human population density, (5) increased human travel and goods transport, (6) changes in living, agricultural and farming habits (e.g. land use) and (7) reduction of resources in the life cycle of mosquitoes by interventions (e.g. source reduction of aquatic habitats). These parameters are all environment-borne, i.e. parameter values are first and foremost dependent on the environmental and climatic conditions, and mosquitoes have to adapt to (e.g. temperature, blood and nectar availability, breeding site availability, etc.). Table 3 summarises what information needs to be considered for IMS surveillance.

**Table 3:** Main characteristics of environmental parameters to be considered for IMS surveillance

**Parameters**	**Information provided**	**Strengths**	**Weaknesses**	**Data collection methods and equipment**
Breeding sites typology, distribution and productivity	Information answers the following questions: where do the mosquitoes breed, what is the relative productivity of the different breeding site types, and what is the geographic distribution throughout the territory?	Good support in the ecological understanding of IMS; identification of targets for IMS control	Requires skilled technicians; high cost	GIS and field data collection
Temperature geo-distribution and trend over the year	Indicates the suitable period for activation of surveillance; feeds the model for IMS risk of establishment and MBD risk assessment; correlates with IMS longevity and vectorial capacity; explains behavioural changes of vector	Data usually available in good detail	Site specific weather data could not be obtained from local weather stations	Data from weather stations usually available locally
				Field-collected data based on portable weather station
Precipitation distribution	Informs the model for IMS risk of establishment; correlates with the IMS population density; informs the population estimate models	Data usually available	Large local variability is difficult to define	Field-collected weather data
Human population density	Informs the model for IMS risk of establishment; informs MBD risk assessment	Data usually available in good local detail	Human behaviour can also have an impact on IMS and MBD risks but these data are usually not available	Socio-statistical data
Vegetation covering	Suitability of the area for colonisation and dispersal	Data usually available in good local detail (CORINE data set)	Requires proficient GIS technicians	Remote sensing data
				Satellite imagery
Human land use in relation to water-keeping habits	Suitability of the area to be colonized; types of water recipients and land cover to be described in terms of larval breeding sites (potential, availability) and energy resources	Data usually available for public areas, but need to be correlated with specific IMS requirements	Private areas difficult to assess; requires time-consuming research	Remote sensing data
				Satellite imagery
				GIS field data collection
Quality and efficacy of IMS control measures	Informs the models for cost-effectiveness estimates; evaluates control methods efficacy/effectiveness (including community participation); resistance management	Ensures independent quality control for IMS control programmes	Requires independent, objective and science based evaluation, as well as skilled technicians	Internal evaluation
				External evaluation

Human population distributions, urbanisation, and movement are the key behavioural factors in most IMS-transmitted diseases because they are related to the global spread of MBD (introduction, reintroduction, circulation) and increase exposure to bites by infected mosquitoes. The world's population is almost equally divided between urban and rural dwellers, and two thirds of Europe's population now live in urban areas, with a similar proportion for the rest of the world projected for 2050 [92]. This trend, which is likely to continue for the foreseeable future, may dramatically enhance the reproduction potential of container-breeding IMS by providing more hosts and habitats. The predicted substantial growth of urban and peri-urban agriculture will also create new breeding sites for IMS as well as influence the distribution of domestic and wild animals.

In order to obtain spatio-temporal perspectives, environmental data collection and analysis should be carried out when there is a high risk of introduction of IMS to an area. In the case of IMS establishment over a wide area, the crucial environmental parameters to be considered are the density, typology, productivity and distribution of breeding sites. These parameters provide key information needed to calculate population abundance, estimate the spread of IMS, and assess the risk of MBD transmission.

### Larval breeding sites and mosquito control

The density and quality of larval breeding sites are directly related to the landscape (natural) and human population (cultural) characteristics in a particular environment (urban, semirural, rural). The success of IMS colonisation of a territory depends to a large degree on the availability and density of breeding sites, and if IMS are introduced, the absence/presence of breeding sites will prevent/favour establishment [93].

Larval breeding sites may be identified and classified based on their characteristics and their productivity for a defined IMS. This can be performed by inspection of breeding sites and collection of mosquitoes (with a dipper or an aquatic net), applying a larval or pupal index (the mean number of larvae/pupae per container type) [94]. Despite the use of simple equipment, this task needs to involve highly trained and skilled professionals with profound knowledge of both the targeted environment and the IMS behaviour (adult oviposition habits, larval and pupal defensive behaviour, etc.). This requires a high level of manpower but effort invested is indispensable for the proper application of control measures.

The attractiveness of potential breeding sites to ovipositing mosquito females is affected by many factors, including the types of water containers and their locations. In a recent study in Italy [29], catch basins in private and public areas resulted the most productive breeding sites for *Ae. albopictus* among the 10 types checked (catch basins, plant saucers, drums, buckets, tarpaulins, tyres, bathtubs, and assorted containers of three different volumes). The highest number of pupae per premise was found in poorly maintained premises, most often in combination with heavy shade. Interestingly, recent study in Malaysia shows the acquisition of an indoor breeding behavior by *Ae. albopictus* the behavioral change that may lead to increased vectorial capacity [95]. A thorough knowledge of the most productive breeding sites is needed to choose the most appropriate population index and establish which site types should be sampled to provide the best indicators of mosquito population abundance. Control programmes may also directly benefit from information on which larval breeding sites are most effective to target. Finally, it would be helpful to obtain information about the quality and efficacy of all conducted IMS control measures, as this will help to later evaluate cost effectiveness and serves to help justify control campaigns.

Mosquito control methods aim at rendering the environment unsuitable for mosquito breeding by applying versatile control measures (biological, chemical, physical). Methods for the evaluation of IMS control quality and efficacy assess the reduction of larvae/pupae per treated breeding site or the reduction of adult mosquitoes (both to measure efficacy of larval and adult control) [96]. Reduction of juveniles can be assessed based on the same method as described above, except in cases when insect growth regulators (IGR) are used: larvae should then be brought to the laboratory to have the adult emergency rate recorded (IGRs have a much slower mode of action than synthetic chemical insecticides) [74,97]. Presence and reduction of adult mosquitoes can be estimated by comparing the number of mosquitoes that are sampled with an adult trap (e.g. number of females/trap/night) or with human bait catches (e.g. number of females/person/15 min) before and after the treatment. For a reliable assessment of the reduction level, untreated plots with mosquito abundance similar to the treated area should be selected and the same method of sampling/trapping applied. Mosquito abundance is best monitored three days before and three days after the treatment because of likely variations in the number of adult mosquitoes (Petrić *et al*., unpublished). Oviposition traps can be used to assess treatment efficacy in case of *Ae. albopictus*. In addition to the assessment of the efficacy of applied measures, a quality check of the control method and procedure may be performed, preferably by an independent external team, in order to review the quality of the performance of the control measures (choice of treatment sites and methods, quality of the performance itself, resistance management, prevention of environmental, health impact, etc.).

### Climate and other global change

National communication reports from most European countries referring to the United Nations Framework Convention on Climate Change (UNFCCC) emphasise a need for the development of climate change mitigation and adaptation strategies. In the area of infectious disease, a key adaptation strategy will be the improved surveillance of MBDs, supplemented by research on whether and how MBDs are influenced by meteorological patterns and climate change. Additional interdisciplinary research on interactions with other risk factors would also be helpful.

The drivers of meteorological and climate change are also of growing international and European-focused interest [98,99]. Projected increases in air temperature are predicted to have an impact on poikilotherm species (whose body temperature depends on the ambient temperature), including the insects that pose a threat to human health. The responses of IMS to these changes (in addition to physiological changes such as the potential for increased vectorial capacity) could lead to an expansion of colonised areas and the invasion of new sites or render some infested areas unsuitable in the future. Increased background temperature due to large urbanization could favour mosquito breeding and, along with higher air temperatures, shorten extrinsic incubation periods, e.g. for the urban IMS vector *Ae. aegypti* and *Ae. albopictus*[47,100]. Recent studies showed that diurnal temperature fluctuations may influence more than expected the extrinsic cycle of the pathogen especially in sub-optimal temperature condition [101]. The overall pattern of the current studies on MBD suggests expanded ranges for disease incidence.

Drivers for the emergence of infectious diseases also include human demographics (e.g. the growth of megacities), international movement of people (travellers and refugees), the smuggling of wildlife, the trade of animals and goods (e.g. trade in used tyres and certain ornamental plants) and various other aspects of globalisation [102]. Increased trade and travel promote the transport of IMS eggs in goods and IMS adults in vehicles, as well as pathogens in infected travellers. Human movement is a critical behavioural factor underlying observed patterns of MBD spread because movement determines exposure to vectors, i.e. bites from infected IMS and transmission of pathogens [103]. Reproduction number of 1.3 is estimated for dengue when exposure is assumed to occur only in the home, as opposed to 3.75 when exposure occurs at multiple locations, e.g. during visits to markets and friends [104]. Interestingly, the model predicted little correspondence between vector abundance and estimated R_0_ when movement is taken into account.

### Meteorological parameters

The observed dispersal of a given species also depends on the weather conditions during the dispersal phase and the characteristics of the locality. In urban areas, important factors include the vegetation type, its abundance and distribution; and the shape and position of buildings, squares, and main roads [44,89,105].

Low relative humidity, high temperatures, and intense solar radiation negatively influence the female biting activity [106], the mean flight distance and reduce the dispersion homogeneity of the males [13]. In hot and dry summer conditions, male mosquitoes showed reduced dispersal and sought shade. Temperature (seasonal averages, altitudinal variation) and precipitation (quantity, seasonal distribution pattern that influence water management habits of the human population) are crucial factors defining the risk of establishment of IMS in an area and should be included in every risk modelling process [96]. Local meteorological parameters should be taken into account, especially in countries with a wide range of temperatures (with different regional climates and a broad altitudinal range). In such countries, monitoring and recording of meteorological parameters are of crucial importance to understand spread and other aspects of IMS vector populations. For example, four years after the introduction in Montenegro, *Ae. albopictus* was recorded in the town Andrijevica at 720–850 mASL, the highest altitude reported until 2005 in Europe [83].

Historical records of temperature and other meteorological data are available for many locations. These databases should be extensively used for the analysis of the IMS populations. However, portable meteorological stations are useful for more precise measurements at locations that are far from the main monitoring points of national meteorological institutions. Medium resolution satellite imagery (e.g. Terra satellite) may also provide valuable meteorological data [96].

### Urban habitats

All IMS currently threatening Europe are container breeders closely connected to urban and peri-urban habitats, where both human and animal hosts are plentiful. Peri-urbanisation occurs when urban regions begin to permeate into neighbouring rural regions and urban development is by far the most rapidly expanding land use change in Europe, rapidly continuing at 0.5 to 0.7% per year, which is more than ten times higher than any other land use change [107].

The adoption of urban lifestyles in rural regions, and likewise rural activities such as farming in urban areas, has driven the growth of peri-urban agriculture, merging the agricultural markets of both settings. Peri-urban agriculture is increasingly being recognised by public health professionals, urban planners, community organisations, and policymakers as a valuable tool for economic development, preservation or production of green space, and improvement of food security [108]. The benefits are many in the context of climate adaptation, economic alleviation and self-sustenance, but urban agriculture also presents challenges for human and animal health, including the increase of IMS breeding sites and the hazard of zoonotic diseases.

Reduction of aquatic habitats (breeding sites) through environmental management mitigates MBD transmission and the emergence of host-seeking mosquitoes, and by increasing the amount of time required for vectors to locate oviposition sites [76]. This applies especially when aquatic habitats are scarce and the mosquito’s flight ability is limited (which is true for all IMS threatening Europe). However, the results of source reduction on mosquito oviposition have largely been neglected in the evaluations of environment management programmes. The characterisation and mapping of breeding sites in urban environments from the beginning of the colonisation is very useful for both entomological and epidemiological surveys and should not be ignored. Mapping can be done at a very high spatial resolution (up to 1 metre spatial resolution) using satellite data [109]. Several satellites carrying sensors with dedicated electro-magnetic channels could be used to characterise urban habitats, a potentially relevant factor when implementing control measures and efficiency assessments. Elimination of larval habitats in 300 m diameter could result in 66% average reduction in MBD incidence compared with 22% for the corresponding conventional interventions [110]. Therefore, source reduction might not, as previously thought, require coverage of extensive areas and that the distance to human homes can be used for habitat targeting.

In addition, dispersal of IMS in an urban environment is not random, and it may be possible to maximise vector control by taking into account ecological factors (e.g. flight corridors) that affect the direction of the flight of female mosquitoes [44].

## Conclusions

Although MBDs represent a far higher burden in tropical than in temperate regions, there have always been both endemic and epidemic autochthonous MBD in Europe. Concern is now rising as both vectors and pathogens are increasingly being introduced by international travel and trade. In addition to arboviruses, IMS may transmit dirofilarial worms in Europe. Numbers of autochthonous infections, though still low, appear to be increasing. Assessing and managing the risk of introduced MBD that have become established in Europe is now a necessity and should also become a priority, in particular in countries where vectors are established. The recent notification of autochthonous chikungunya and dengue fevers cases in Europe shows its vulnerability to these diseases in areas where the vector, the invasive mosquito *Ae. albopictus* or *Ae. aegypti* is present. Strengthening surveillance of exotic mosquito species such as *Ae. albopictus*, *Ae. aegypti*, *Ae. japonicus*, *Ae. koreicus*, *Ae. atropalpus*, and *Ae. triseriatus* in areas at risk of importation or spread of mosquitoes and risk of arbovirus transmission is therefore essential. This is particularly important in the context of changes in eco-systems, human behaviour, and climate, which might allow an increase of vector populations and virus amplification. Guidance on customised surveillance methods that encourage the European countries governments to collect appropriate data on IMS in the field is considered necessary. Early detection of IMS increases the opportunity for appropriate and timely response measures and therefore MBD prevention. In addition, in areas where IMS have become established, permanent monitoring of their abundance and expansion is needed for timely risk assessment of pathogen transmission to humans. Optimal scenario would be to harmonise surveillance methods and information records at European level so that data from different countries/areas can be compared over time. In addition, the arrival of IMS in cities can affect public perception of the effectiveness of control programmes already in place. Controlling a mosquito that breeds in containers around human settlements and potentially generates MBD transmission is completely different to controlling a myriad of nuisance marshland mosquitoes that occasionally reach the cities but transmit few benign MBD. Besides that, although monitoring of mosquito populations and environmental parameters is often neglected by authorities when planning the budget, these are essential for improving prevention and control of IMS and MBD.

## Abbreviations

BI: Breteau index; CI: Container index; CHIKV: Chikungunya virus; ECDC: European centre for disease prevention and control; FR: Flight range; HBI: Human blood index; HI: House index; HLC: Human landing collection; IGR: Insect growth regulators; IMS: Invasive mosquito species; MAX: Maximum distance travelled; MBD: Mosquito borne diseases; MDT: Mean distance travelled; MED: Egg density index; MRR: Mark-release-recapture; NBC: Number of bites per citizen; OI: Ovitrap index; PDS: Pupal demographic surveys; PHI: Number of pupae per hectare; PPI: Number of pupae per premise; PPP: Pupae per person; R0: Basic disease reproduction number; SIT: Sterile insect technique; TP: Trap positivity index; UNFCCC: United Nations framework convention on climate change; WNV: West Nile virus.

## Competing interests

The authors declare that they have no competing interests.

## Authors’ contributions

DP and FS conceived the review and wrote the first draft of the manuscript. All authors contributed to the improvement of the manuscript, and read and approved the final version.
